# Short‐Term Exposure of Bisphenol A Deteriorates the Quality of Rabbit Milk by Impairing Milk Fat Synthesis

**DOI:** 10.1002/fsn3.4561

**Published:** 2024-11-22

**Authors:** Jia Hao, Shaohui Beng, Zifeng Ma, Hongmei Xu, Ting Yang, Qiman Yang, Yunduan Wang, Wenhui Zheng, Yisha Ma, Shuo Zhang, Liangde Kuang, Wei Fu

**Affiliations:** ^1^ College of Animal & Veterinary Sciences Southwest Minzu University Chengdu China; ^2^ Sichuan Animal Sciences Academy Chengdu China; ^3^ Key Laboratory of Qinghai‐Tibetan Plateau Animal Genetic Resource Reservation and Utilization, Ministry of Education Southwest Minzu University Chengdu China; ^4^ Key Laboratory of Animal Science of National Ethnic Affairs Commission of China Southwest Minzu University Chengdu China

**Keywords:** bisphenol A, lactating rabbits, lipid synthesis, milk metabolome

## Abstract

This study aimed to investigate the effects of short‐term exposure of Bisphenol A (BPA) on the growth and lactation performance, blood parameters, and milk composition of lactating rabbits and explore its potential molecular mechanisms. Eight lactating rabbits with similar body weight were selected and randomly divided into the experimental group (BPA) and the control group (Ctrl). The group BPA was orally administered 80 mg/kg/day BPA on the 15th day postpartum, while the group Ctrl received a corresponding volume of vehicle. Blood and milk samples were collected after 7 days treatment. The results showed that short‐term ingestion of BPA did not obviously alter the body weight, feed intake, or milk yield of the lactating rabbits. ELISA assays indicated that BPA did not significantly affect the plasma levels of glutathione peroxidase (GSH‐Px), superoxide dismutase (SOD), creatinine (CRE), alanine aminotransferase (ALT), aspartate aminotransferase (AST), uric acid (UA), and urea. Utilizing untargeted metabolomics, we first depicted the metabolomic profile of rabbit milk, and identified 277 differential metabolites (DMs), with 141 DMs upregulated (e.g., BPA, and its metabolites including Cetirizine N‐oxide) and 136 DMs downregulated (e.g., Oleamide, Tiglic acid, PC O‐38:4) in the group BPA. KEGG analysis revealed that the DMs were mainly enriched in pathways comprising fatty acid metabolism, fatty acid degradation, and phosphatidylinositol signaling system, emphasizing the effect of BPA on milk fat metabolism. Hence, we established the BPA‐induced MAC‐T model, and the results showed that BPA significantly reduced cell viability and impacted lipid synthesis, as evidenced by reduced lipid droplets (BODIPY and Oil Red O staining) and decreased expression of genes related to lipid synthesis (e.g., *PPARγ*, *ACACA*, *LPL*). In summary, we first drew the metabolomic profile of rabbit milk and confirmed that short‐term BPA exposure impacted mammary lipid synthesis, thereby reducing the milk quality of lactating rabbits and providing fundamental data for resolving the toxicological mechanisms of BPA on mammal lactation.

## Graphical abstract

This study investigates the detrimental effects of short‐term Bisphenol A (BPA) exposure on the milk quality of lactating rabbits, particularly focusing on its impact on milk fat synthesis, using metabolomics and in vitro cell culture approaches.

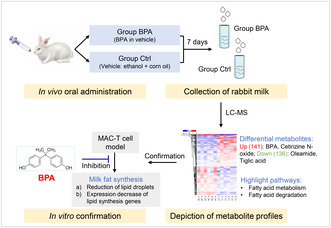

## 1 Introduction

Environmental estrogens (EEs) contaminate habitats all around the world (Adeel et al. 2017). These substances, originating from industrial chemicals, pesticides, and consumer products, enter human and animal bodies through various routes and potentially cause adverse health effects (Adeel et al. 2017; Qie et al. 2021; Wojnarowski et al. 2021). The primary hazards of EEs include disrupting the endocrine system, thereby affecting the normal functions of the reproductive and nervous systems (McLachlan 2016). Bisphenol A (BPA) is a typical representative of EEs (Morgan et al. 2017).

BPA is widely used in the production of polycarbonate plastics and epoxy resins, with a global production of up to 10 million tons annually (Mishra, Goel, and Shankar 2023). These raw materials are used to manufacture various consumer goods, including water bottles, food containers, and medical devices. Despite its wide range of applications, the potential hazards of BPA cannot be ignored. BPA owns an estradiol‐like structure, which allows it to bind to estrogen receptors (ERs) and naturally trigger cellular responses. Therefore, BPA interferes with normal cellular signaling pathways, such as the oxytocin signaling pathway (Witchey, Fuchs, and Patisaul 2019), and affects human health even at low concentrations (Vandenberg et al. 2007). The effects of BPA on the nervous and reproductive systems include damage of neuronal activity, dysfunction of HPG axis, and infertility (Santoro et al. 2019). There is evidence that exposure to low doses of BPA can lead to changes in mammary gland development and morphology, and promote the proliferation of breast cancer cells (Santoro et al. 2019; Segovia‐Mendoza et al. 2020). Diabetes, obesity, hypertension, and other cardiovascular or cardiometabolic diseases are major risk factors for global mortality, and related studies have shown that blood and urine BPA levels are positively correlated with the increased risk of cardiovascular or cardiometabolic diseases (Kang, Asai, and Toita 2023). Serum BPA levels before dialysis in diabetic patients are significantly higher than in non‐diabetic patients (Turgut et al. 2016); a case–control study of 439 pairs of hypertensive patients and healthy control participants indicated that BPA exposure is positively correlated with the risk of hypertension (Jiang et al. 2021). Due to the above toxicities of BPA, major countries around the world have implemented restraining orders on BPA use, prohibiting its application in infant products (Fox, Versluis, and van Asselt 2017). However, breastfeeding mothers may still come into contact with BPA products, and potentially transfer BPA to their offspring through breast milk, making infant exposure to BPA unavoidable.

Milk is synthesized and secreted primarily by mammary epithelial cells through a complex physiological process strictly regulated by various hormones. Milk contains numerous nutrients, as well as immune and antibacterial components, including milk fat, milk protein, and lactose, which play critical roles in the growth and development of neonates (Andreas, Kampmann, and Mehring Le‐Doare 2015). The primary energy source in breast milk is lipids, accounting for 40%–55% of the total energy (Koletzko et al. 2001). Studies have found that lipids in breast milk (e.g., milk fat globule membrane, MFGM) provide immune protection for the fetus, preventing invasive infections on mucous membrane surfaces (Isaacs, Litov, and Thormar 1995). Milk proteins are essential nutrients, which support infant growth and brain development, promote nutrition absorption, provide antibacterial properties, and enhance immunomodulatory activities (Lönnerdal 2004; Molinari et al. 2012); Human milk oligosaccharides (HMOs), the primary carbohydrates in lactose, can facilitate the colonization of gut microbiota, promote the development of the gastrointestinal barrier, and regulate intestinal immune function (Cheng et al. 2021; Donovan and Comstock 2016; Sakanaka et al. 2019).

BPA contamination is widespread and detectable in human blood, urine, and milk (Vandenberg et al. 2010). Remarkably, a study on BPA detection in global milk samples revealed an average BPA concentration of 1.4 ng/mL (Iribarne‐Duran et al. 2022). Even in infants not exposed to environmental BPA, urinary BPA concentrations range from 1.2 to 4.4 μg/L (Mendonca et al. 2014). As a persistent lipophilic chemical, ingestion of BPA during lactation tends to accumulate in the mammary glands of mammals, affecting milk quality. Perinatal exposure to BPA leads to a delay in mammary gland differentiation at the end of pregnancy in rats, and the levels of α‐lactalbumin and β‐casein in milk are reduced (Kass et al. 2012). Furthermore, perinatal exposure to low doses of BPA alters the composition and ratio of fatty acids in milk, and the synthesis of milk fat globules is also affected (Altamirano et al. 2015). Increasing evidences suggest that BPA exposure inhibits mammary gland development and differentiation (Perrot‐Applanat et al. 2018), and raises the risk of mammary gland abnormalities in females (Koual et al. 2020), including mastitis and breast cancer (Ayyanan et al. 2011; Durando et al. 2007; Leonel et al. 2021). However, the effects of perinatal BPA exposure are not limited to female individuals; they also cause irreversible damage to the fetus. Studies have shown that the offspring of female rodents exposed to BPA during the perinatal period have significantly higher body weights than normal offspring (Altamirano et al. 2015), and their normal liver metabolic profiles are also disrupted (Meng et al. 2019). Currently, despite many studies having explored the potential mechanisms of BPA‐induced reproductive toxicity as an estrogen‐like hormone, the impact of BPA on lactation and milk quality in lactating rabbits is still lacking.

Based on the aforementioned findings, the present study used lactating rabbits as the model to investigate the impacts of short‐term BPA exposure on milk composition and quality by analyzing the differences in milk metabolites between the group Ctrl and BPA using untargeted metabolomics. Furthermore, using the mammary epithelial cell line MAC‐T as an in vitro model, we further confirmed the effects of BPA on milk fat synthesis. This research laid the foundation for dissecting the impacts of BPA on lactation and milk quality in mammals encompassing human beings.

## 2 Materials and Methods

### 2.1 Animal Management and Experimental Design

The lactating rabbits used in the experiment were Shuxing No. 1 meat rabbits, raised with commercial feed (Jiajie, meat rabbit compound feed (Grade II)) produced by Sichuan Xinye Meilin Feed Co. Ltd. The feed composition included corn (Grade I), soybean meal (43%), wheat (Grade II), wheat bran, alfalfa pellet, dicalcium phosphate, limestone (calcium content 38%), sodium chloride, L‐lysine hydrochloride (98.5%), vitamin A, vitamin D, vitamin E, basic copper chloride (Cu 50%), manganese sulfate (Mn 31.8%), ferrous sulfate heptahydrate, and zinc sulfate (Zn 34.5%). After parturition, the maternal rabbits were randomly divided into two groups, each with four replicates, and each replicate consisted of one mother rabbit nursing eight kits. The mothers normally nursed the neonates for 2 weeks, and the gavage experiment was initiated on the 15th day post‐parturition. In the experiment group (BPA), the lactating rabbits were orally administered BPA at a dosage of 80 mg/kg (body weight)/day for 7 days; the control group (Ctrl) received a corresponding volume of a mixture of ethanol and corn oil (vehicle). For drug preparation, 80 mg of BPA powder was dissolved in 400 μL of anhydrous ethanol, then corn oil was added to make up the total volume of 2 mL. This solution was then drawn into a syringe and injected into the mouth of the lactating rabbits in the group BPA (without the needle), ensuring that the entire mixture was ingested. Gavage was performed once daily at 9:00 AM.

### 2.2 Production Performance Measurement

The body weight of the lactating rabbits was recorded on the day 0, 3, and 7 of the experiment. The weight of the offered feed and the remaining feed each day were measured and recorded to calculate the daily feed intake. The method for measuring the milk yield of the lactating rabbits involved the following steps: (1) removing the neonates from the cage of lactating rabbits at the previous night (21:00 PM), (2) weighing the body weight of the lactating rabbits at the next morning (9:00 AM) and recording as M1, (3) returning the kids to the cage of lactating rabbits correspondingly for breast‐milk feeding (within 20 min), (4) measuring the body weight of the lactating rabbits and recording as M2. The milk yield of lactating rabbits was calculated as: M1 − M2.

### 2.3 Milk Sample Collection

On day 7th of the experiment, we restrained the lactating rabbits and collected the milk by manually squeezing the fourth pair of nipples. The milk was gathered into centrifuge tubes, with each mother rabbit yielding 1 to 2 mL of milk. The milk was immediately frozen in liquid nitrogen for subsequent metabolite analysis.

### 2.4 Blood Sample Collection and Measurement

On the 7th day of the experiment, blood samples were collected from the auricular vein of the lactating rabbits. The blood was allowed to clot at room temperature for 2 h, then centrifuged at 800 rpm for 20 min to collect the upper layer of serum, which was then stored in a −80°C freezer. Serum indicators were measured using the commercial ELISA kits (Suzhou Greace Biotech Co. Ltd) according to the instructions, including the superoxide dismutase (SOD) activity assay kit by WST‐8 method (G0101W), glutamate oxaloacetate transaminase/aspartate aminotransferase (GOT/AST) kit (G0424W), glutathione peroxidase (GSH‐Px) assay kit (G0204W), uric acid content (uricase method) assay kit (G1202W), urea content (enzyme method) assay kit (G1201W), creatinine (CRE) content (Creatininase method) assay kit (G1204W), and glutamate pyruvate transaminase/alanine aminotransferase (GPT/ALT) kit (G0423W).

### 2.5 Cultivation and Differentiation of MAC‐T Cells

MAC‐T cells were cultured and passaged in DMEM (Thermo, Massachusetts, USA) supplemented with 10% FBS (Fetal Bovine Serum, Newzerum, Christchurch, New Zealand) and 1% P/S (Penicillin–Streptomycin, Hyclone, USA). The culture conditions were maintained at 37°C with 5% CO_2_, and the medium was changed daily. When cells reached 80% confluence, the differentiation medium was added, consisting of the above growth medium plus 10 ng/mL PRL (MCE, New Jersey, USA), 10 ng/mL EGF (MCE), 5 μg/mL transferrin (TF, Aladdin, Shanghai, China), 50 μg/mL insulin (INS, MCE), and 10 μg/mL hydrocortisone (HC, MCE), to induce differentiation for 3 days.

### 2.6 Cell Viability Assay

Cell viability was assessed using the CCK‐8 assay (Biosharp, Hefei, China). Firstly, approximately 2 × 10^3^ cells were seeded into each well of a 96‐well plate and incubated for 24 h to allow attachment. After all cells had adhered to the plate, 100 μL of differentiation medium containing various concentrations of BPA was added to the PRL‐cultured cells, and the cells were incubated for 3 days. Subsequently, 10 μL of enhanced CCK‐8 solution was added to each well under darkness, and the plates were incubated at 37°C for 2 h. Finally, the absorbance at 450 nm was measured using a microplate reader.

### 2.7 Real‐Time Quantitative Polymerase Chain Reaction (RT‐qPCR)

Firstly, the total RNA was extracted using Total RNA Isolation Reagent (Biosharp, Hefei, China) according to the instructions. The cDNA was then synthesized using a reverse transcription kit (Vazyme, Nanjing, China) with the following reaction systems and conditions: 1) 4 μL of 4 × gDNA wiper Mix, 1 μg of total RNA, and ddH_2_O up to 16 μL; The reaction condition: 42°C for 2 min. 2) Adding 4 μL of 5 × qRT SuperMix to the above mixture; the reaction condition: 50°C for 15 min, 85°C for 5 s. Quantitative analyses of target genes were performed using the RT‐qPCR kit (Vazyme), with a reaction system consisting of 5 μL of 2 × ChamQ SYBR qPCR Master Mix, 0.5 μL of forward primer, 0.5 μL of reverse primer, 1 μL of cDNA template, and 3 μL of ddH_2_O. The RT‐qPCR protocols consisted of 95°C, 30 s for pre‐denaturation; 95°C, 10 s for denaturation, and 60°C, 15 s for extension, with 40 cycles; followed by a melting curve from 65°C to 95°C. The relative expression level of genes was calculated using the $2^{\Delta \Delta C_{t}}$ method as per the previous description (Fu et al. 2024). Based on the gene sequences from NCBI (https://www.ncbi.nlm.nih.gov/), quantitative primers for the target genes and the internal reference gene (*β‐ACTIN*) were designed using the NCBI online tools (Table 1).

**TABLE 1:** Primers for RT‐qPCR.

Gene name	Sequence	Lengths (bp)
*PPARγ*	F: GATAGGTGTGATCTTAACTGTCGGAT R: CGCTAACAGCTTCTCCTTCTCG	150
*LPL*	F: TCCTGGAGTGACCGAATC R: ACAAGGCAGCCACGAGTT	212
*ACACA*	F: GTGTGAAGTTCCCTCAGGCTCTTA R: GTCTGAGCAGATATCCACTTCCA	278
*β‐ACTIN*	F: TCAGCAAGCAGGAGTACGATGA R: ATCCTGAGTCAAGCGCCAAA	164

### 2.8 BODIPY and Hoechst Dual Staining

Lipid droplets and nuclei in MAC‐T cells were stained using BODIPY (Beyotime, Shanghai, China) and Hoechst (Solarbio, Beijing, China) dyes, respectively. When the cell differentiation was complete, cells were washed thrice with serum‐free medium and incubated with BODIPY and Hoechst working solutions at 37°C in the darkness for 30 min. Cells were then rinsed in the same way to reduce background interference. Fluorescence images were observed and captured using an Olympus TH4‐200 microscope (Tokyo, Japan) with a 10× objective lens (NA 0.70). Subsequently, fluorescence quantification was performed using Image J software (NIH, USA).

### 2.9 Oil Red O Staining

Lipid droplets in differentiated MAC‐T cells were stained and qualitatively analyzed using Oil Red O (Solarbio). Initially, the culture medium was discarded, and the cells were washed once with PBS. Then, 4% paraformaldehyde (PFA) was added to fix the cells at room temperature for 30 min. After that, the cells were washed once with PBS to remove residual PFA. Subsequently, Oil Red O working solution (0.5% w/v Oil Red O in 60% v/v isopropanol) was added to the cell culture plates and incubated at 37°C for 30 min. After staining, the cells were washed with PBS to remove excess working solution, leaving a small amount of PBS in the plates to prevent dispersion of the lipid droplets. Bright‐field images were captured using an Olympus TH4‐200 microscope. Finally, Oil Red O dye was extracted from the stained adipocytes using 100% isopropanol, and the dye concentration was quantitatively analyzed by measuring the optical density (OD) at 510 nm.

### 2.10 Untargeted LC–MS Metabolomics

To meet the sample size requirements for untargeted metabolomics analysis, equal volumes of milk samples from both the group Ctrl and BPA were mixed and then divided into six equal parts for subsequent testing and analysis. The samples were sent to Shanghai Lingen Biotechnology Co. Ltd. (Shanghai, China) for untargeted metabolomics analysis using LC–MS/MS. The main steps were described as follows.

#### 2.10.1 Metabolite Extraction

100 μL of milk sample was placed into an EP tube, added 400 μL of 80% methanol aqueous solution, and vortexed well, following an ice bath for 5 min. The samples were then centrifuged at 15,000 *g* for 20 min under 4°C. Next, we diluted a certain volume of the supernatant with mass spectrometry grade water to adjust the methanol content to 53% and centrifuged again with the same condition. Finally, the supernatant was collected and preserved.

#### 2.10.2 LC–MS/MS Analysis

The liquid chromatography–tandem mass spectrometry (LC–MS/MS) analysis was conducted using a Q ExactiveTM HF‐X series mass spectrometer (Thermo Fisher Scientific) coupled with a Vanquish UHPLC chromatography system (Thermo Fisher Scientific). Samples were injected onto a Hypersil Gold column (C18) with a 12‐min linear gradient elution at a flow rate of 0.2 mL/min. In the positive mode, the mobile phases consisted of solvent A (0.1% formic acid) and solvent B (methanol). For the negative mode, the mobile phases were solvent A (5 mM ammonium acetate, pH 9.0) and solvent B (methanol).

#### 2.10.3 Preprocessing of Raw Data

We used Compound Discoverer 3.3 (CD3.3) for spectral processing and database searching to conduct preliminary screening of each metabolite. Briefly, we set retention time deviations of 0.2 min and mass deviations of 5 ppm for peak alignment across different samples, followed by setting mass deviation of 5 ppm, a signal intensity deviation of 30%, a signal‐to‐noise ratio of 3, minimum signal intensity, and aggregated ions for peak extraction.

#### 2.10.4 Metabolite Database Annotation

Metabolites were annotated and categorized to understand their basic classification and function. The databases used included the Kyoto Encyclopedia of Genes and Genomes (KEGG), Human Metabolome Database (HMDB), and LIPID MAPS.

#### 2.10.5 Metabolite Data Analysis

Based on the metabolite abundance results of each sample, we performed principal component analysis (PCA) and orthogonal partial least squares discriminant analysis (OPLS‐DA) on the metabolite abundance data of all samples. Using the VIP (Variable Importance in the Projection) values from the OPLS‐DA model, combined with the *p*‐values from independent sample *t*‐tests, we identified differential metabolites [VIP ≥ 1, *p* ≤ 0.05 and fold change (FC) ≥ 1.2 || FC ≤ 1/1.2]. These differential metabolites were visualized using volcano plots, lollipop plots, and radar plots, which also helped filter out metabolites of interest. KEGG pathway enrichment analysis was used to find metabolic pathways most correlated with the differences in metabolites.

### 2.11 Statistical Analysis

Statistical analyses were performed using the SPSS 18.0 software package (SPSS Science, Chicago, IL, USA). Experimental data were subjected to *t*‐test and ANOVA analyses, with a significance threshold set at *p* < 0.05. Graphs were generated using GraphPad Prism 8 software (GraphPad, Santiago, USA). Data were presented as mean ± standard deviation (SD).

## 3 Results

### 3.1 Effects of Short‐Term BPA Exposure on Growth and Lactation Performance of Lactating Rabbits

**FIGURE 1:**
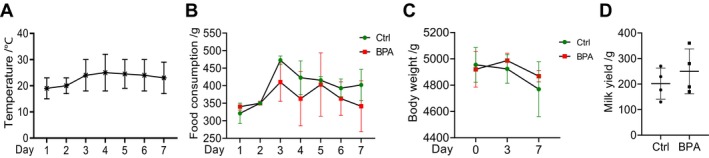
Effects of short‐term BPA exposure on growth and lactation performance of lactating rabbits. (A) The average temperature statistics during the experimental period; (B) Food consumption of the lactating rabbits in the group Ctrl and BPA (*n* = 4); (C) Body weight statistics of the lactating rabbits in the group Ctrl and BPA (*n* = 4); (D) Lactation performance of the lactating rabbits in the group Ctrl and BPA (*n* = 4).

The animal experiment in the present study was conducted under an average temperature range of 19°C–25°C, effectively avoiding stress responses due to excessively high or low temperatures (Figure 1A). Statistical analysis of the food consumption revealed a reduced trend in BPA‐treated rabbits (*p* > 0.05) (Figure 1B). Meanwhile, BPA exposure did not result in a significant decrease in the body weight of the lactating rabbits (*p* > 0.05) (Figure 1C). Similarly, BPA did not markedly alter the lactation performance of the lactating rabbits (*p* > 0.05) (Figure 1D).

### 3.2 Effects of Short‐Term BPA Exposure on Serum Parameters in Lactating Rabbits

**FIGURE 2:**
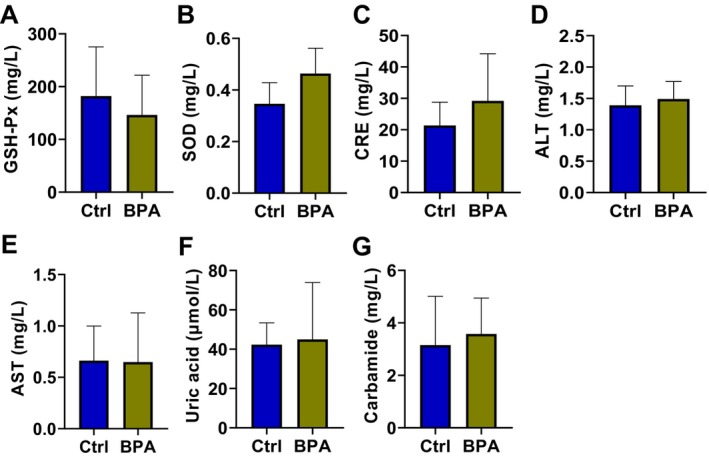
Effects of short‐term BPA exposure on serum parameters in lactating rabbits. (A–G) The ELISA assay detected the levels of Glutathione peroxidase (GSH‐Px) (A), Superoxide Dismutase (SOD) (B), Alanine Aminotransferase (ALT) (C), Aspartate Aminotransferase (AST) (D), Creatinine (CRE) (E), Uric Acid (UA) (F), and urea (G) in the serum of group Ctrl and BPA (*n* = 4).

Through the ELISA assay of the serum parameters, we further evaluated the effects of short‐term BPA exposure on the antioxidant capacity (Glutathione peroxidase [GSH‐Px] and Superoxide Dismutase [SOD]), liver function (Alanine Aminotransferase [ALT] and Aspartate Aminotransferase [AST]), and kidney function (Creatinine [CRE], Uric Acid [UA], and Urea) in lactating rabbits. Our results indicated that BPA did not significantly change these serum parameters in the lactating rabbits (Figure 2A–G).

### 3.3 Untargeted Metabolomics Analysis of the Effects of Short‐Term BPA Exposure on Rabbit Milk Metabolic Characteristics

**FIGURE 3:**
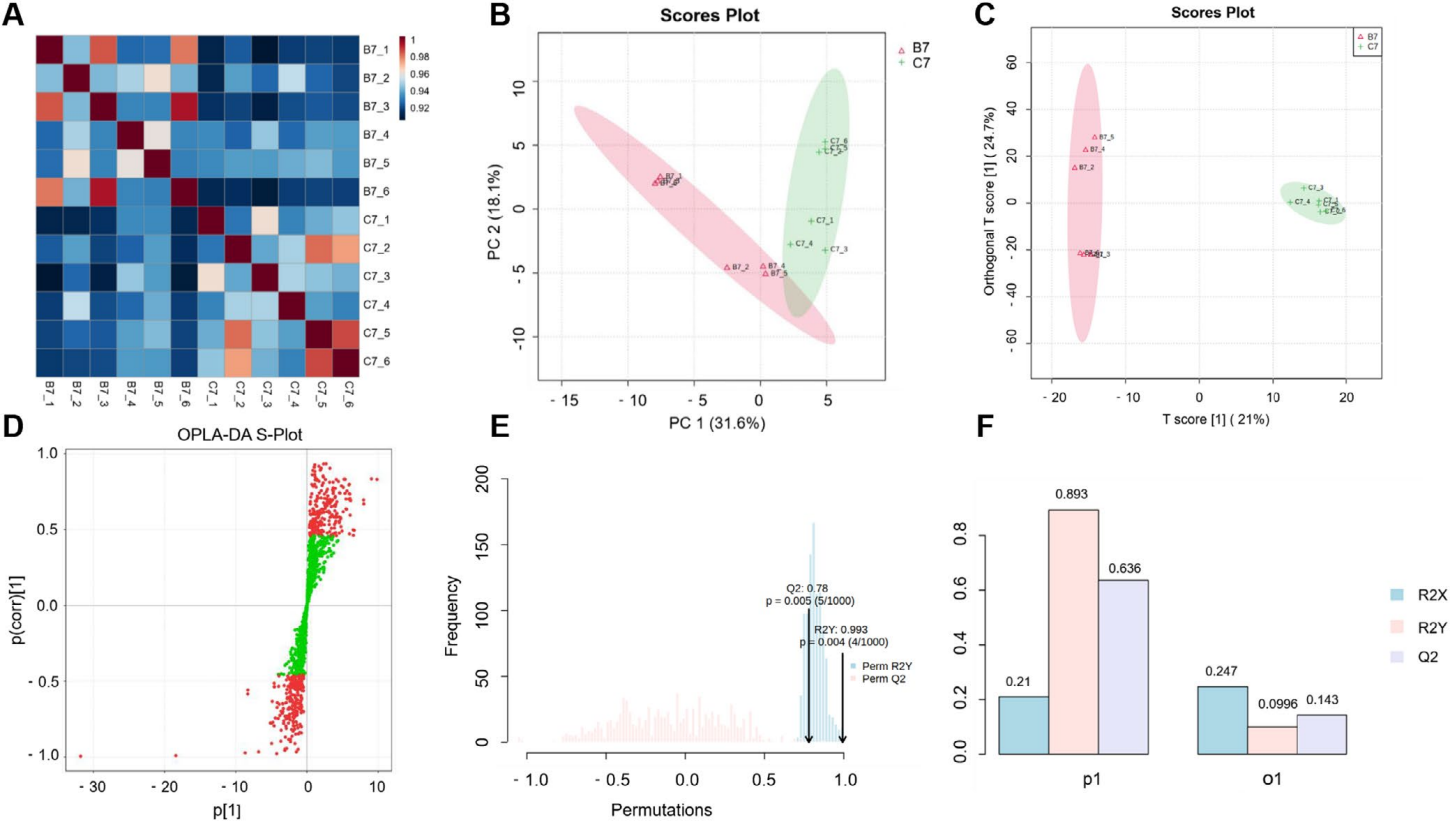
Analysis of sample correlation and group differences. (A) Heatmap of sample correlation; (B) PCA score plot; (C) OPLS score plot; (D) S‐plot; (E) Permutation test plot; (F) OPLS‐DA model plot.

Using untargeted metabolomics, we analyzed the effects of short‐term BPA exposure on the metabolic characteristics of milk in lactating rabbits. Firstly, we calculated the Pearson correlation coefficients among each sample in the group Ctrl and BPA and found that the correlation coefficients of each sample were > 0.9, indicating a high degree of correlation among the samples (Figure 3A). We then performed principal component analysis (PCA) according to the metabolite abundance results. Accordingly, the results showed that PC1 and PC2 accounted for 31.6% and 18.1% of the variance, indicating excellent biological replication (Figure 3B). Combined with the orthogonal partial least squares discriminant analysis (OPLS‐DA) results, we further indicated a significant difference in milk metabolite composition between the group Ctrl and BPA (Figure 3C). Additionally, under both positive and negative ion modes, the R2Y and Q2 values were 0.993 and 0.78, respectively, both > 0.5, indicating that the model fit accurately without overfitting (Figure 3D–F). These results suggested that there was a significant difference in metabolite composition between the group Ctrl and BPA, indicating that BPA altered the metabolic profile of milk in lactating rabbits.

In the present experiment, a total of 1263 metabolites were detected under both positive (Pos) and negative (Neg) ion modes (Table S1). Notably, the top five metabolites detected in both the group Ctrl and the group BPA were the same, but their rankings differed, being citric acid, oleamide, creatine phosphate, DL‐carnitine, and N‐[2‐chloro‐6‐(trifluoromethoxy)phenyl]‐2,2‐dimethylpropanamide, respectively. Among them, only oleamide showed a significant decrease in the group BPA.

**FIGURE 4:**
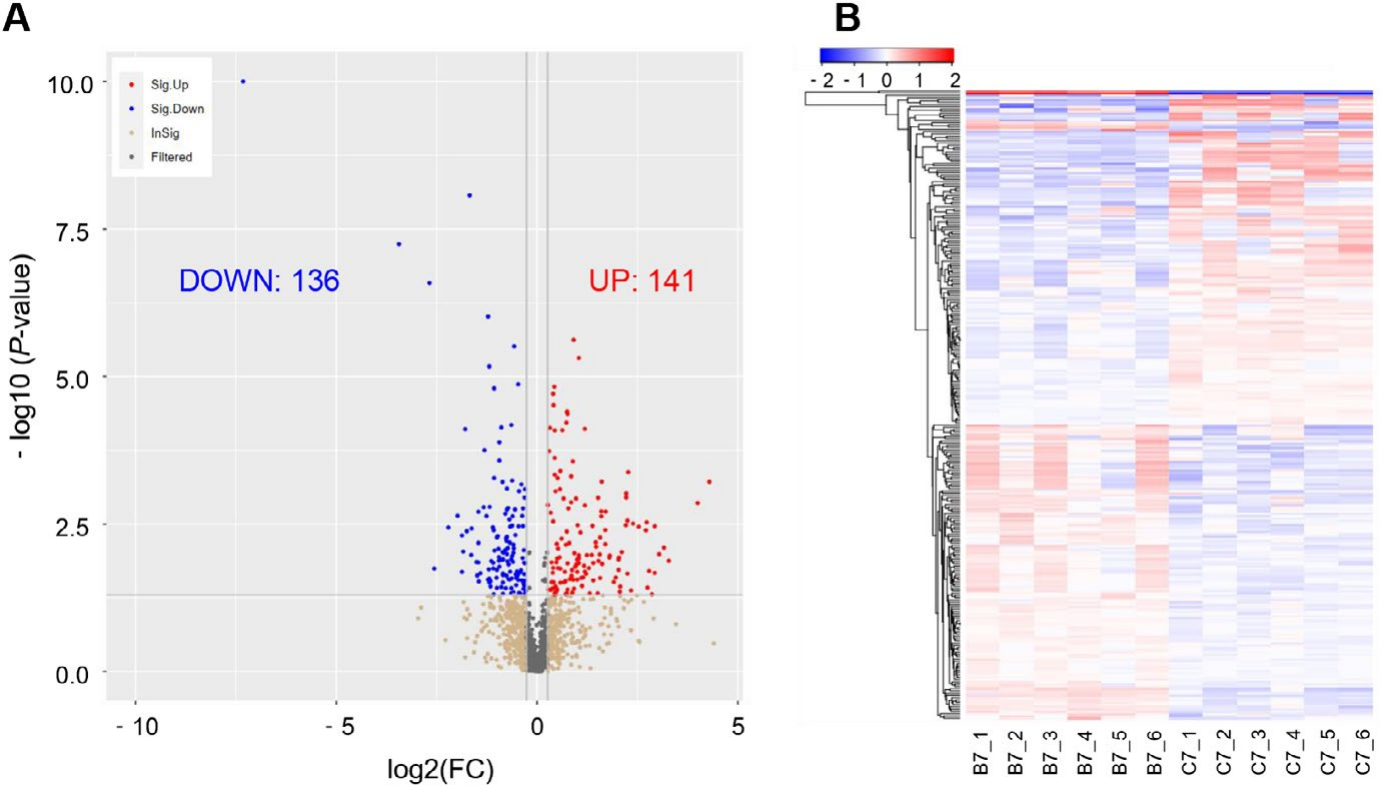
Differential metabolites (DMs) analysis of rabbit milk between group Ctrl and BPA. (A) Volcano plot of DMs between group Ctrl and BPA; (B) clustering heatmap of DMs between group Ctrl and BPA.

Additionally, we conducted a differential analysis on all metabolites detected between group Ctrl and BPA. Accordingly, we generated a volcano plot under the combined positive and negative ion modes, and identified a total of 277 differential metabolites (DMs), of which 141 were significantly upregulated and 136 were significantly downregulated in group BPA compared to group Ctrl (Figure 4A and Table S2). Meanwhile, these DMs were visualized using a clustering heatmap (Figure 4B).

**FIGURE 5:**
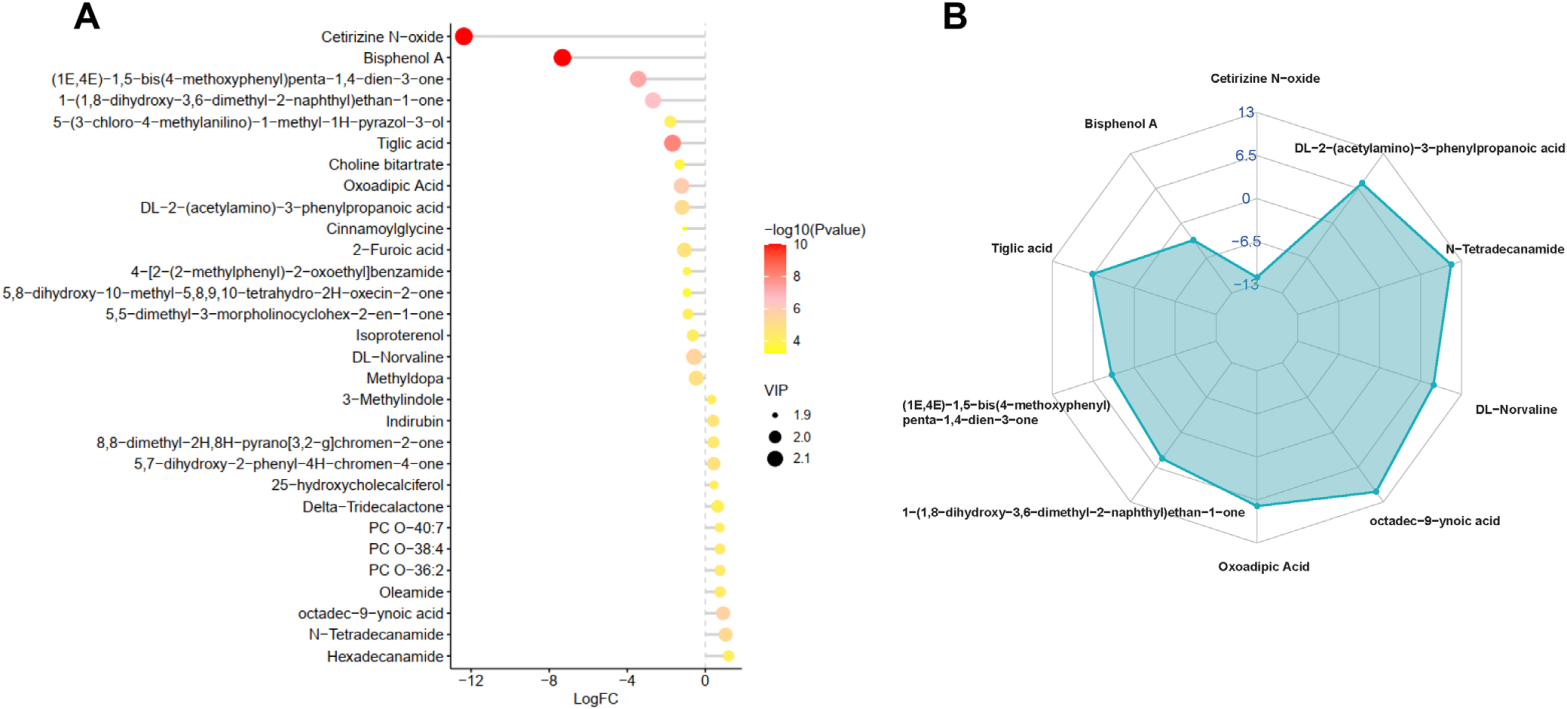
DMs Analysis according to VIP value and radar chart. (A) VIP plot of the top 30 DMs; (B) radar chart of DMs.

Based on the Variable importance in the projection (VIP) values, we analyzed the top 30 DMs, including 17 upregulated DMs and 13 downregulated DMs. Interestingly, the top 10 DMs were all significantly upregulated in the group BPA, including Cetirizine N‐oxide, BPA, Tiglic acid, DL‐Norvaline, Oxoadipic Acid, Methyldopa, and 2‐Furoic acid (Figure 5A). The radar chart highlighted the top 10 DMs contributing most significantly to the distinction between the two groups, with BPA and its derivative Cetirizine N‐oxide being the most significantly upregulated in the group BPA, while lipid‐related substances such as Tiglic acid and Oxoadipic Acid were sharply downregulated (Figure 5B). Milk fat is an essential nutritional source for infants and young children, and various lipid substances are indispensable for the synthesis of milk fat. Overall, these results indicated that BPA disrupted the synthesis and metabolism of various lipid substances in organisms, including the synthesis of milk fat. The disruption of lipid metabolism by BPA might have significant implications for maternal health, milk quality, and fetal development.

**FIGURE 6:**
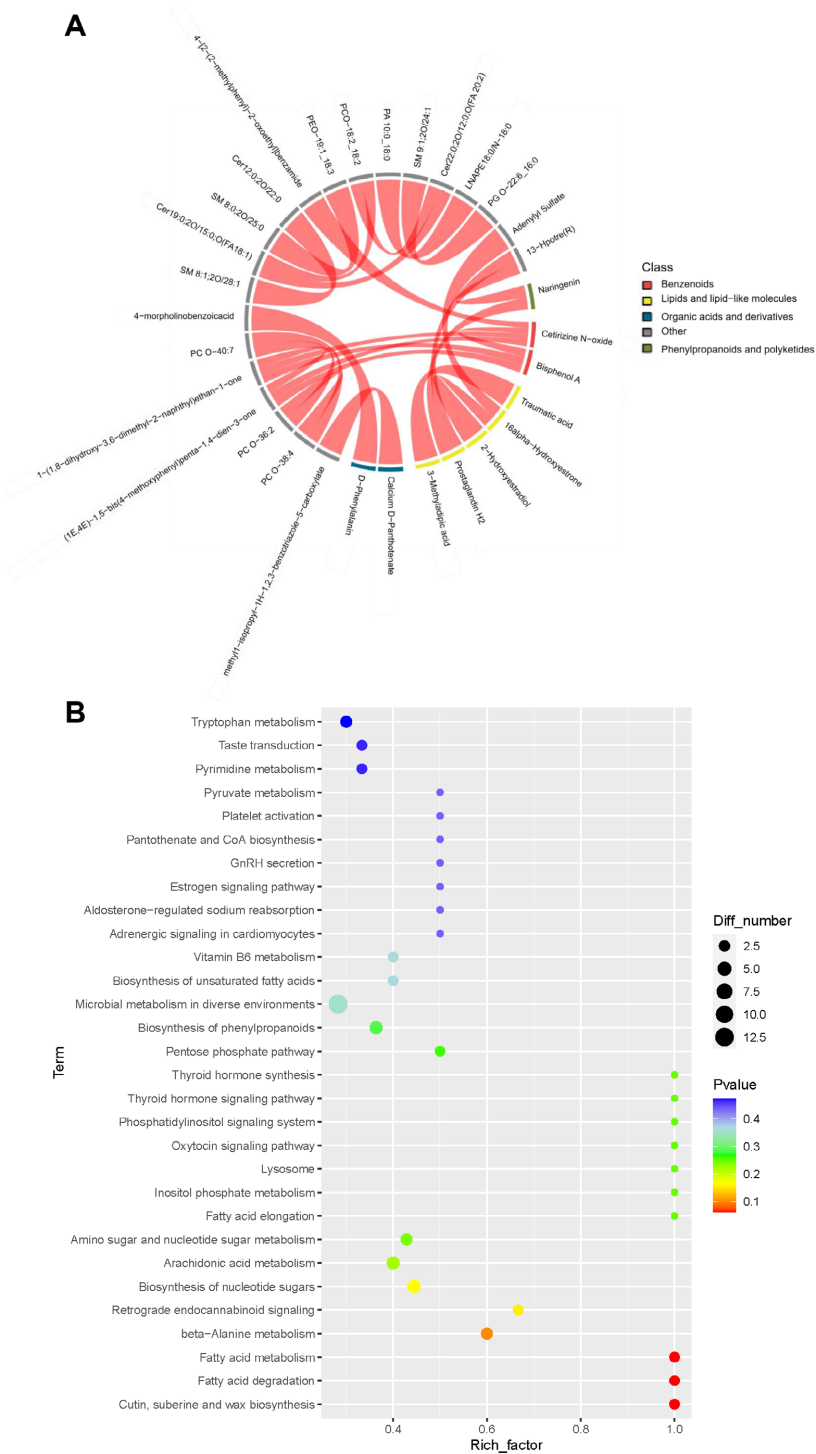
KEGG Pathway Analysis of DMs. (A) Chord diagram of DMs; (B) KEGG pathway enrichment chart.

The chord analysis of DMs visually demonstrated the interconnections among various DMs. BPA and cetirizine N‐oxide exhibited connecting chords with phosphatidylcholine‐like and ketone metabolites, suggesting that BPA ingestion might influence the metabolism of phosphatidylcholine and ketone substances within lactating rabbits. Additionally, lipids and lipid‐like molecules also exhibit associations with other metabolites (Figure 6A). Further comprehensive KEGG pathway enrichment analysis was performed on these DMs, ranked by *p*‐value from the smallest to largest one. The main metabolic pathways identified were fatty acid metabolism, fatty acid degradation, and fatty acid elongation (Figure 6B and Table S3). These results indicated that BPA exposure has the most significant impact on lipid metabolism.

These results further suggested that short‐term BPA exposure disrupts the metabolism related to lipid synthesis in the milk of lactating rabbits, altering the composition of milk fat and thereby impairing the quality of the milk.

### 3.4 Confirming the Effect of BPA on Milk Fat Synthesis Using In Vitro MAC‐T Model

**FIGURE 7:**
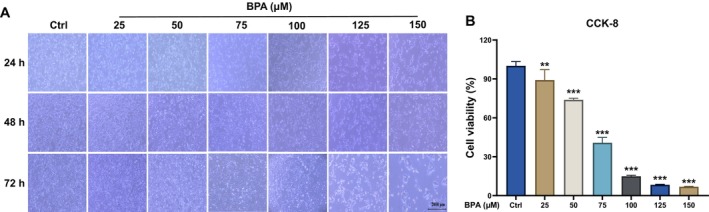
The effect of BPA on cellular status and cell viability in MAC‐T cells. (A) Bright‐field images of PRL‐cultured MAC‐T cells incubated with different concentrations of BPA (0, 25, 50, 75, 100, 125, and 150 μM) at different time‐points (0, 24, 48, and 72 h); (B) The effect of different concentrations of BPA (0–150 μM) on the cell viability of PRL‐cultured MAC‐T after 72 h treatment (*n* = 4). **p < 0.01, ***p < 0.001.

Based on the above results of metabolomics, we further utilized an in vitro MAC‐T model to evaluate the toxic effects of BPA on differentiated mammary epithelial cells. Firstly, different concentrations of BPA (0, 25, 50, 75, 100, 125, 150 μM) were supplemented to the medium at the onset of MAC‐T differentiation. After 72 h incubation, the bright‐field observation revealed that BPA significantly affected cell morphology and reduced cell numbers in a dose‐dependent manner (Figure 7A). Next, using the CCK‐8 assay to assess the impact of BPA on MAC‐T cell viability, we found that cell viability significantly decreased accompanied by the concentration increase of BPA compared to the group Ctrl (*p* < 0.01) (Figure 7B). On the basis of these results, we selected 25, 50, and 100 μM BPA to implement the subsequent experiments. These findings indicated that BPA significantly affected cell morphology and reduced cell viability.

**FIGURE 8:**
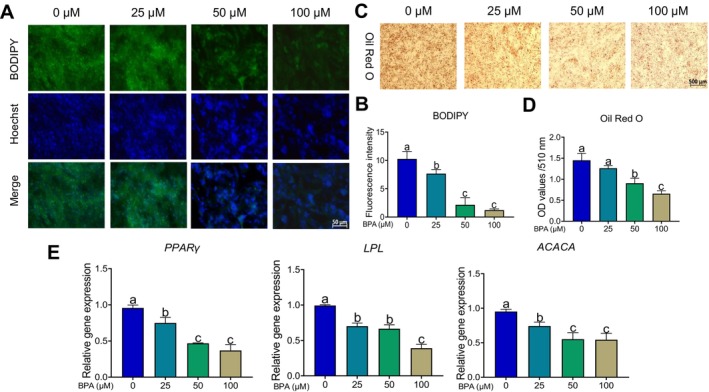
The effect of BPA on milk fat synthesis and secretion in PRL‐cultured MAC‐T cells. (A–B) Representative images (A) and quantitative analysis (B) of BODIPY staining of PRL‐cultured MAC‐T cells treated with different concentrations of BPA (*n* = 3). The y‐axis indicates fluorescence intensity. (C–D) Representative images (C) and quantitative analysis (D) of Oil Red O staining of PRL‐cultured MAC‐T cells treated with different concentrations of BPA (*n* = 3). The y‐axis represents the absorbance at 510 nm. (E) mRNA levels of key genes involved in milk fat synthesis (e.g., *PPARγ*, *LPL*, *ACACA*) in PRL‐cultured MAC‐T cells treated with different concentrations of BPA. Different letters on each column meant significant differences.

Subsequently, we treated the PRL‐cultured MAC‐T cells with varying concentrations (0, 25, 50, 100 μM) of BPA for 72 h and assessed the milk fat synthesis and secretion. The results of Oil Red O and BODIPY staining indicated that with the increase of BPA concentration, the lipid droplet secretion of MAC‐T cells was significantly inhibited (Figure 8A–D). Additionally, we evaluated the mRNA levels of genes involved in milk fat synthesis (e.g., *PPARγ*, *LPL*, *ACACA*). We found that BPA significantly inhibited the expression of these genes (Figure 8E) (*p* < 0.05). These findings suggested that BPA impeded the milk fat synthesis in mammary epithelial cells, which was positively correlated with BPA concentration.

## 4 Discussion

Research on rabbit lactation is relatively limited, and no studies have reported the effects of BPA on the growth, health, lactation, and milk quality of lactating rabbits. In this study, gavage ingestion of BPA did not significantly alter the body weight, feed intake, or milk performance of lactating rabbits (Figure 1). Additionally, liver function, kidney function, and antioxidant capacity were not drastically affected (Figure 2), which might be due to the short duration of BPA intake.

Milk, secreted by the mammary glands, is the most important nutritional source for the healthy growth and development of infants (“Breastfeeding and the use of human milk,” Section on Breastfeeding 2012). The mammary gland is a complex secretory organ, with the lactation process mediated by highly branched lobular units and milk transported through the ductal network within the mammary tissues (Kumar et al. 2023; Nguyen et al. 2018). Mature milk mainly consists of water (about 87%), lactose (7%), lipids (3.8%), and proteins (1.0%) (Martin, Ling, and Blackburn 2016). Mammary gland development and differentiation during lactation are highly susceptible to hormonal interference. As a typical xenoestrogen, BPA can disrupt mammary epithelial cells with short‐term intake, affecting normal lactation and posing a risk of inducing breast cancer (Ruiz et al. 2022). Using untargeted metabolomics, we detected a significant presence of BPA and its metabolite Cetirizine N‐oxide in the milk of BPA‐treated rabbits, indicating that BPA intake of rabbits accumulated in the mammary glands via blood circulation and was secreted into the milk. This result supported our hypothesis that BPA intake affected milk quality.

Furthermore, we utilized untargeted metabolomics to depict the metabolic profile of rabbit milk for the first time. Among the detected metabolites, Citric acid was identified as the most abundant metabolite in both the group Ctrl and BPA. It is well‐documented that Citric acid is an important organic weak acid involved in the tricarboxylic acid (TCA) cycle, a critical pathway for the oxidative breakdown of organic substances and energy production in cellular metabolism. There is limited research on the role of citric acid in milk, but its participation in the TCA cycle suggests that metabolic disturbances of citric acid may impede the energy metabolism of mammary epithelial cells. Additionally, Oleamide, a significant metabolite that was notably decreased in the group BPA in our study, is an endogenous fatty acid amide that can be de novo synthesized in the nervous system of mammals (Mendelson and Basile 2001). It is also one of the most common long‐chain fatty acid amides found in breast milk and is known to promote the development of infant's brain and nervous system by increasing the number of neurons in the hippocampus and enhancing synaptic plasticity (Tao et al. 2022). A decrease in Oleamide levels in milk may affect the early neurodevelopment and maturation of offspring. Meanwhile, maternal BPA exposure is associated with neurological and behavioral deficits in childhood. Studies have indicated that BPA can be transferred from the mother to the fetus, leading to structural and functional abnormalities in the fetal cortex through the activation of autophagy, thereby altering the morphogenesis and synaptic function of the offspring's cerebral cortex (Jiang et al. 2024; Lee et al. 2021). As a common environmental contaminant, the potential impact of BPA on animal husbandry should not be overlooked. BPA contamination may alter the composition of metabolites in milk, affecting the quality of animal products and posing a threat to food safety. Furthermore, considering the potentially disruptive effects of BPA on mammary epithelial cells, long‐term exposure to BPA may have adverse effects on human health, particularly on the development of infants.

Our further analysis of the results suggested that BPA significantly altered the metabolic patterns of milk (Figure 3), and affected lipid synthesis in the mammary glands (Figures 4, 5, 6). Milk fat is the primary energy supplier in milk and is a crucial source of essential nutrients, such as polyunsaturated fatty acids (PUFA), Fat‐soluble vitamins, and Bioactive compounds (Koletzko 2016). Human milk lipids consist of 35%–40% saturated fatty acids, 45%–50% monounsaturated fatty acids, and 15% polyunsaturated fatty acids (Grote et al. 2016). Our analysis found that BPA significantly reduced the levels of lipids such as Oleamide, Hexadecanamide, Palmitic acid, and Phosphatidylcholine in rabbit milk, thereby lowering milk quality. The nutrients in milk are vital for the early brain development of infants (Cusick and Georgieff 2016). Hexadecanamide, another fatty acid amide, is known for its neuroprotective properties, although its mechanism of action remains unclear (Patel et al. 2020). Additionally, hexadecanamide can limit inflammation and repair blood‐milk barrier integrity, effectively alleviating mastitis (Bao et al. 2023). Palmitic acid is a major saturated fatty acid in milk, accounting for 17%–25% of total fatty acids (Demmelmair and Koletzko 2018). Additionally, Palmitic acid in breast milk facilitates calcium absorption in infants, promoting bone development and beneficial gut microbiota colonization (Demmelmair and Koletzko 2018). Phosphatidylcholine is the predominant phospholipid that forms the bilayer structure in the cell membranes of mammals and is also an essential component of the milk fat globule membrane (Deeth 1997). Disruptions or alterations in its synthesis or metabolic pathways can significantly affect the fluidity and movement of membranes within the cells (Fagone and Jackowski 2013). BPA significantly downregulated phosphatidylcholine expression, suggesting that short‐term BPA intake interferes with phosphatidylcholine synthesis and metabolism, hindering the formation of the milk fat globule membrane. To summarize, short‐term exposure to BPA significantly reduced the levels of hexadecanamide, palmitic acid, and phosphatidylcholine in milk, potentially inhibiting the neural development of offspring. More seriously, BPA altered the composition of milk fat and reduced the quality of milk lipids, which could be detrimental to the overall development of the progeny.

This study enriched the identified differential metabolites using the KEGG database (Figure 6B), with the main enriched pathways being Fatty acid metabolism, Fatty acid degradation, Arachidonic acid metabolism, Fatty acid elongation, and Retrograde endocannabinoid signaling. The KEGG enrichment results indicated that DMs were primarily enriched in the lipid metabolism pathways. Hence, our research emphasis was specifically directed toward the fatty acid metabolism pathway. Fatty acids are crucial constituents for the structural development of the fetal brain and retina, exerting a direct influence on fetal growth and maturation (Yang and Lin 2021). BPA interferes with fatty acid metabolism, a process that includes the synthesis, breakdown, and conversion of fatty acids. Disruptions in this metabolic pathway have been implicated in numerous pathological conditions, such as obesity (Machate et al. 2020), cardiovascular diseases (Frayn, Fielding, and Karpe 2005), and diabetes mellitus (Calder 2015), and may impede fetal development (Heath, Klevebro, and Wood 2022). Fatty acid metabolism is integral to cellular energy homeostasis. While no conclusive evidence currently establishes a direct link between fatty acid catabolism and the quality of breast milk, the energy requirements of lactation suggest that the capacity for fatty acid breakdown in the mother could indirectly influence the energetic content of breast milk, subsequently affecting the energy provision and developmental trajectory of the infants.

It is noteworthy that ingestion of BPA results in modifications to the fatty acid metabolic pathways and exerts effects on the nervous and endocrine systems of maternal rabbits. Endocannabinoids modulate a spectrum of physiological functions and behaviors, including motor control, appetite regulation, and nociception, via the activation of cannabinoid receptors present in the central nervous system (Koch 2017; Sañudo‐Peña et al. 2000; Starowicz, Malek, and Przewlocka 2013). Furthermore, our findings indicated that acute BPA exposure disrupts thyroid hormone synthesis in maternal rabbits. While existing literature implicates perinatal exposure to endocrine disruptors, such as dichloroacetic acid, in the impairment of neonatal thyroid hormone levels and subsequent inhibition of neurodevelopment, there remains a dearth of research substantiating analogous detrimental effects of BPA on infants (Cordier et al. 2015). Our experimental data prompt the hypothesis that BPA may exert adverse effects on thyroid hormone levels in both maternal and infant populations. Further investigation is warranted to elucidate the potential risks associated with BPA exposure during the perinatal period. Based on the above metabolomics data, we further validated the toxic effects of BPA on lipid synthesis in mammary cells using the in vitro mammary epithelial cell model (MAC‐T) (Figures 7 and 8). The results from the MAC‐T cell experiments were consistent with the KEGG pathway enrichment analysis, both indicating that BPA affected the synthesis and metabolism of milk lipids. Studies have reported that BPA interferes with the estrogen pathway by binding to estrogen receptors, inhibiting prolactin release, and thereby suppressing milk secretion (Kasper et al. 2016). Additionally, perinatal BPA exposure impairs mammary gland functional differentiation and alters milk lipid composition in rats (Altamirano et al. 2015). Our MAC‐T cell experiments showed that BPA significantly inhibited the growth and viability of mammary epithelial cells, suppressed lipid droplet formation, and downregulated the expression of genes related to lipid synthesis. In short, our study demonstrated that BPA exposure significantly altered milk lipid metabolism and impacted mammary gland function, providing new insights into the potential health implications of environmental chemicals in lactation.

As we mentioned in the introduction section, BPA, as a typical environmental estrogen, has been widely present in the living environment of human beings and animals since the last century. Due to the major economic entities around the world, including the European Union, the United States, and China, explicitly restricting the use of BPA, especially in the manufacture of baby products, the harm BPA brings to humans and infants has been reduced to a certain extent. However, in many underdeveloped areas, BPA is widely used due to its low cost, and the potential harm of BPA to humans is still non‐negligible. Infants and young children have a much lower metabolic capacity for exogenous substances than adults, who can indirectly ingest BPA through maternal milk, making the risk of BPA exposure and accumulation even greater (Nahar et al. 2013). Our results uncovered that BPA exerted inhibitory and disruptive effects on the quality of rabbit milk, particularly impacting the synthesis of milk fat. Additionally, studies have reported that BPA can be detected in the tissues of several different species of marine and freshwater organisms, with the highest concentration in wild fish reaching up to 13,000 ng/g (Corrales et al. 2015). The specific impacts of BPA exposure on aquatic wildlife include an increased rate of embryonic deformities, decreased sperm quality, and delayed ovulation, among other reproductive and developmental issues (Lahnsteiner et al. 2005; Pastva et al. 2001; Sohoni et al. 2001). While these results underscored the necessity for further research to clarify the underlying mechanisms, they also accentuated the adverse effects of BPA exposure with respect to infant nutrition and development. Future research should concentrate on investigating the impact of BPA on the health of offspring and devising potential intervention strategies to ameliorate these effects.

## 5 Conclusions

We depicted the metabolomic profile of rabbit milk for the first time. Meanwhile, our results demonstrated that short‐term exposure to BPA did not change the growth performance, milk daily yield, and oxidative capability of lactating rabbits, however, significantly altered the metabolomic pattern of rabbit milk, especially the synthesis of milk fats, which were validated via the in vitro MAC‐T model.

## Author Contributions

**Jia Hao:** formal analysis (equal), investigation (equal), methodology (equal), validation (equal), writing – original draft (equal). **Shaohui Beng:** investigation (equal), methodology (equal), software (equal), validation (equal), writing – original draft (equal). **Zifeng Ma:** data curation (equal), formal analysis (equal), methodology (equal), validation (equal), writing – original draft (equal), writing – review and editing (equal). **Hongmei Xu:** data curation (equal), formal analysis (equal). **Ting Yang:** data curation (equal), formal analysis (equal). **Qiman Yang:** data curation (equal), formal analysis (equal), writing – original draft (equal). **Yunduan Wang:** data curation (equal), formal analysis (equal). **Wenhui Zheng:** data curation (equal), formal analysis (equal). **Yisha Ma:** data curation (equal), formal analysis (equal). **Shuo Zhang:** data curation (equal), formal analysis (equal). **Liangde Kuang:** data curation (equal), formal analysis (equal), funding acquisition (equal), validation (equal), writing – original draft (equal), writing – review and editing (equal). **Wei Fu:** data curation (equal), formal analysis (equal), funding acquisition (equal), validation (equal), writing – original draft (equal), writing – review and editing (equal).

## Conflicts of Interest

The authors declare no conflicts of interest.

## Supporting information


Tables S1–S3.

## Data Availability

The data used to support the findings of this study are available from the corresponding authors upon request.
